# Effects of Static Stretching With High-Intensity and Short-Duration or Low-Intensity and Long-Duration on Range of Motion and Muscle Stiffness

**DOI:** 10.3389/fphys.2020.601912

**Published:** 2020-11-20

**Authors:** Taizan Fukaya, Ryosuke Kiyono, Shigeru Sato, Kaoru Yahata, Koki Yasaka, Remi Onuma, Masatoshi Nakamura

**Affiliations:** ^1^Institute for Human Movement and Medical Sciences, Niigata University of Health and Welfare, Niigata, Japan; ^2^Department of Rehabilitation, Kyoto Kujo Hospital, Kyoto, Japan; ^3^Department of Physical Therapy, Niigata University of Health and Welfare, Niigata, Japan

**Keywords:** static stretching, range of motion, stretch tolerance, shear elastic modulus, stretching duration, stretching intensity

## Abstract

This study investigated the effects of static stretching (SS) delivered with the same load but using two protocols – high-intensity and short-duration and low-intensity and long-duration – on range of motion (ROM) and muscle stiffness. A total of 18 healthy students participated in the study. They randomly performed high-intensity and short-duration (120% and 100 s) or low-intensity and long-duration (50% and 240 s) SS. Outcomes were assessed on ROM, passive torque at dorsiflexion ROM, and shear elastic modulus of the medial gastrocnemius before and after static stretching. The results showed that ROM increased significantly at post-stretching compared to that at pre-stretching in both high-intensity and short-duration [+6.1° ± 4.6° (Δ25.7 ± 19.9%)] and low-intensity and long-duration [+3.6° ± 2.3° (Δ16.0 ± 11.8%)]. Also, the ROM was significantly higher at post-stretching in high-intensity and short-duration conditions than that in low-intensity and long-duration. The passive torque at dorsiflexion ROM was significantly increased in both high-intensity and short-duration [+5.8 ± 12.8 Nm (Δ22.9 ± 40.5%)] and low-intensity and long-duration [+2.1 ± 3.4 Nm (Δ6.9 ± 10.8%)] conditions, but no significant differences were observed between both conditions. The shear elastic modulus was significantly decreased in both high-intensity and short-duration [−8.8 ± 6.1 kPa (Δ − 38.8 ± 14.5%)] and low-intensity and long-duration [−8.0 ± 12.8 kPa (Δ − 22.2 ± 33.8%)] conditions. Moreover, the relative change in shear elastic modulus in the high-intensity and short-duration SS was significantly greater than that in low-intensity and long-duration SS. Our results suggest that a higher intensity of the static stretching should be conducted to increase ROM and decrease muscle stiffness, even for a short time.

## Introduction

Static stretching (SS) increases range of motion (ROM) and decreases muscle stiffness (Morse et al., 2008; Nakamura et al., 2014) and is commonly performed in sports and rehabilitation. Increases in ROM and decreases in muscle stiffness could be important for sports performance and activities of daily living (Mulholland and Wyss, 2001; Hemmerich et al., 2006) and might influence risk of muscle strain injury (Witvrouw et al., 2003). Therefore, many researchers have investigated the effectiveness of various methods of SS on ROM and muscle stiffness.

Recently, Apostolopoulos et al. (2015) reported that the four parameters associated with the effect of SS on flexibility are intensity, duration, position, and intervention frequency. In particular, previous research reported that a longer stretching duration was more effective in changing ROM and muscle stiffness (Matsuo et al., 2013) and that high-intensity (120% intensity) SS had greater effects on ROM and muscle stiffness (Kataura et al., 2017). Therefore, it is assumed that higher-intensity or longer-duration SS could be more effective in increasing ROM and decreasing muscle stiffness. However, there are no previous studies that have investigated the effect of both different stretching intensities and durations on ROM or muscle stiffness.

Research into the effects of resistance training concluded that total work, which was calculated from training intensity, repetitions, and number of sets, was important for muscle hypertrophy (Burd et al., 2010; Schoenfeld et al., 2019). We assumed that the load of SS could be effective for changes in ROM and muscle stiffness, because the previous studies showed changes in ROM and muscle stiffness were greater with a higher intensity or longer duration of SS. However, a few studies focused on the effect of different intensity and duration of stretching intervention, with the same load in which the stretch intensity and duration variables were inversely manipulated, on ROM and passive stiffness. For instance, Freitas et al. (2015b) investigated the effect of similarly structured SS protocols (90 s with 100% intensity vs. 135 s with 75% intensity vs. 180 s with 50% intensity), they found that higher stretching intensity with short-duration was more effective in increasing ROM, and longer duration with lower intensity was more effective in decreasing passive torque at a given angle. In contrast, when Marchetti et al. (2019) compared the effect of 240 s SS with 50% stretching intensity and 120 s SS with 85% intensity, and they found no significant difference between the two protocols. Thus, there was no consensus about the effect on ROM of SS with the same product of stretch intensity and duration but different intensity and duration. Moreover, to the best of our knowledge, no study has investigated the effect of different SS protocols with the same product of stretch duration and intensity including 120% intensity stretching on muscle stiffness. Thus, this study aimed to investigate the effect of high-intensity and short-duration or low-intensity and long-duration SS with the same load on ROM and muscle stiffness. We hypothesized that higher-intensity and shorter-duration SS would have a greater effect on ROM while lower-intensity and longer-duration SS would have a greater effect on muscle stiffness, because previous studies reported that higher-intensity SS was more effective on ROM and longer-duration SS was more effective in decreasing passive torque at a given angle (Freitas et al., 2015b).

## Materials and Methods

### Experimental Design

A randomized crossover design was used to investigate the effect of the two SS protocols on ROM and muscle stiffness of the dominant leg, which was determined by kicking a ball. The two SS conditions – high-intensity and short-duration stretching (120% intensity and 100 s) and low-intensity and long-duration stretching (50% intensity and 240 s) – were performed randomly with an interval of more than 1 week. Our choice of SS intensity and duration was based on previous research (Freitas et al., 2015b). Dorsiflexion (DF) ROM, passive torque at DF ROM, and shear elastic modulus of the medial gastrocnemius muscle (MG) were measured before (PRE) and after (POST) stretching. At POST, we measured the DF ROM and the passive torque at DF ROM immediately after stretching. After the measurement of the DF ROM and the passive torque at DF ROM, the shear elastic modulus was measured. All measurements were performed within about 3 min after stretching.

### Participants

A total of 18 healthy young adults (males, 11; females, 7) who did not play sports or have a high activity daily participated in the study (age, 21.5 ± 0.5 years; height, 167.3 ± 8.0 cm; and body weight, 61.6 ± 7.8 kg). In previous studies, the effect of SS has not been investigated separately for men and women (Ryan et al., 2009; Kay et al., 2015). Therefore, this study did not separate men and women. Participants were excluded if they had a history of surgery on their back or lower body, lower-extremity contracture, neurological disorders, or if they took hormone or muscle-affecting drugs. Written informed consent was obtained from all participants. In addition, this study was approved by the Ethics Committee at our institution (17677).

### Measurement of Dorsiflexion Range of Motion and Passive Torque at Dorsiflexion Range of Motion

Participants were seated in an isokinetic dynamometer (Biodex system 3.0; Shirley, NY, USA) chair at 0° knee angle (i.e., the anatomical position) and 70° hip flexion to prevent tension at the back of the knee, with adjustable belts over the trunk and pelvis (Nakamura et al., 2020). We adjusted the seat position to prevent the heel raise during passive dorsiflexion, and the ankles of participants were fixed to the footplate by two adjustable belts firmly. Furthermore, we visually confirmed no heel raise during the passive stretching. Then, participants were passively moved the footplate of the dynamometer starting from the ankle at 0° angle to the dorsiflexion angle at the point of feeling of discomfort at 5°/s speed until stopping the dynamometer by activating a safety trigger (Kubo et al., 2001; Morse et al., 2008). The participants were instructed to relax and stop the dynamometer at the point of the feeling of discomfort. DF ROM and passive torque at DF ROM were calculated from the torque-angle curve using Biodex (Matsuo et al., 2013; Nakamura et al., 2020). All participants performed the familiarization session 1–2 weeks before the experimental trials. DF ROM (°) was defined as the maximum dorsiflexion angle. Passive torque at DF ROM (Nm) was defined as passive torque at the point of maximum dorsiflexion (Mizuno et al., 2013). Two trials were performed and the greatest dorsiflexion or passive torque at DF ROM was used for further analysis (Blazevich et al., 2014).

### Measurement of Shear Elastic Modulus of Medial Gastrocnemius Muscle

**Figure 1:**
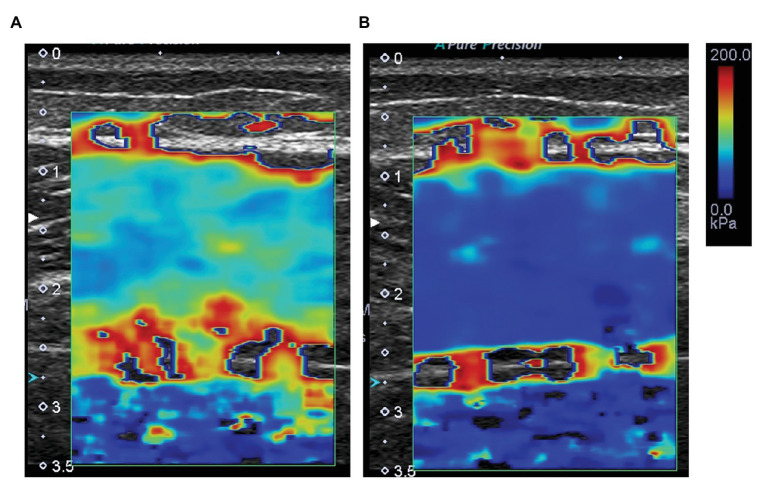
Typical examples of measurement of shear wave speed (Vs) before **(A)** and after **(B)** stretching. The measurement of shear wave speed was set as the region of interest in the area as large as possible in MG, and the average value of the shear wave speed inside this region was obtained. The colored region represents the shear modulus map with the scale right the images.

We used ultrasonic shear wave elastography (Aplio 500, Toshiba Medical Systems, Tochigi, Japan) with a 5–14 MHz linear probe to measure the shear elastic modulus of MG. A previous study showed that the reliability of shear elastic modulus measurements used by the elastography device was very high [ICC (1,1) was 0.952; Sato et al., 2020]. The participants were in similar positions to those used during the measurement of DF ROM. The shear elastic modulus of MG at 10° dorsiflexion was measured at 30% of the lower leg length from the popliteal crease to the lateral malleolus near the point at which the maximal cross-sectional area in the lower leg is located (Nakamura et al., 2014, 2020). Ultrasound image measurements were performed twice in long-axis image of MG. The analysis of shear wave speed in ultrasound images was performed using image analysis software (MSI Analyzer version 5.0, Rehabilitation Science Research Institute, Japan). The measurement of shear wave speed (Vs) was set as the region of interest in the area as large as possible in MG, and the average value of the shear wave speed inside this region was obtained (Figure 1). The shear elastic modulus was calculated as *μ* (kPa) = *ρ*Vs^2^, where *ρ* is muscle mass density (1,000 kg/m^3^). The average value of shear elastic modulus obtained from two ultrasound images was used for analysis.

### Static Stretching Intervention

SS was performed for the plantar flexor muscles in a similar position to the measurement of DF ROM. A stretching intensity of 100% was defined as the DF ROM at PRE. The stretching angle at 120% intensity (high-intensity and short-duration protocol) or 50% intensity (low-intensity and long-duration protocol) was determined by the DF ROM at PRE. The static stretching was performed for one session of 100 s (high-intensity and short-duration condition) or one session of 240 s (low-intensity and long-duration condition). We performed two stretching conditions randomly with an interval of more than 1 week and less than 3 weeks.

### Measurement Reliability

The test-retest reliabilities were investigated for all variables in seven healthy young adults. The calculated intraclass correlation coefficients for the DF ROM, the passive torque at DF ROM, and the shear elastic modulus were 0.97 (95% CI: 0.84–0.99), 0.90 (95% CI: 0.58–0.98), and 0.83 (95% CI: 0.37–0.97), respectively, which indicates that the reliability for all outcome measures was high (Landis and Koch, 1977). Moreover, we calculated the minimal detectable change (MDC) using the standard error of the mean. The MDC of DF ROM, the passive torque at DF ROM, and the shear elastic modulus were 4.4°, 5.7 Nm, 14.2 kPa, respectively.

### Statistical Analysis

We assessed the normality of the data by the Shapiro-Wilk test. This test revealed that the data of the DF ROM was normally disturbed, but the passive torque at DF ROM and the shear elastic modulus of MG were not disturbed. Therefore, the parametric tests were applied to the data of the DF ROM, but the nonparametric tests were applied to the data of the passive torque at DF ROM, the shear elastic modulus, and the relative change for all variables. For DF ROM, we performed a two-way repeated measures ANOVA [time (PRE vs. POST) and conditions (high-intensity and short-duration protocol vs. low-intensity and long-duration protocol)] to analyze the interaction and main effect. Furthermore, we used the Bonferroni *post hoc* test to determine the significant differences between PRE and POST in each protocol. For the passive torque at DF ROM and the shear elastic modulus, Wilcoxon signed-rank test with Bonferroni correction was performed to compare PRE and POST. For the relative change for all variables, Wilcoxon signed-rank test was performed to compare between the conditions. Using the *t*-value and sample size, the effect size (ES) was calculated when the outcome was applied to parametric tests. On the other hand, using *z*-value and sample size, ES was calculated when the outcome was applied to non-parametric tests. The ES classification was set, where *r* < 0.1 was considered trivial, 0.1–0.3 was considered small, 0.3–0.5 was considered moderate, and >0.5 was considered large (Cohen, 1988). All statistical analyses were performed using R2.8.1 (CRAN, freeware), and significance was set at *p* < 0.05. All data are presented as mean ± standard deviation.

## Results

All variables in both protocols are presented in Table 1. The repeated two-way ANOVA indicated a significant interaction effect for DF ROM (*F* = 7.0, *p* = 0.017, *ηp*^2^ = 0.291). The *post hoc* test revealed a significant increase in DF ROM after SS in the high-intensity and short-duration condition (*p* < 0.01, *ES* = 0.85) and the low-intensity and long-duration condition (*p* < 0.01, *ES* = 0.81). Moreover, DF ROM at POST in the high-intensity and short-duration protocol was significantly higher than that in the low-intensity and long-duration protocol (*p* < 0.01, *ES* = 0.70). The relative change in DF ROM was significantly greater in the high-intensity and short-duration condition than the low-intensity and long-duration condition (*p* = 0.038, *ES* = 0.48). Passive torque at DF ROM was significantly increased from PRE to POST in both the high-intensity and short-duration condition (*p* = 0.036, *ES* = 0.55) and low-intensity and long-duration condition (*p* = 0.013, *ES* = 0.62). However, the relative change in passive torque at DF ROM was no significant differences between conditions (*p* = 0.067, *ES* = 0.44). Shear elastic modulus of MG was significantly decreased from PRE to POST in both the high-intensity and short-duration condition (*p* < 0.01, *ES* = 0.88) and low-intensity and long-duration condition (*p* = 0.036, *ES* = 0.55). Moreover, the relative change in shear elastic modulus of MG was significantly greater in the high-intensity and short-duration condition and the low-intensity and long-duration condition (*p* = 0.038, *ES* = 0.49).

**Table 1:** The effects of high-intensity and short-duration or low-intensity and long-duration stretching on DF ROM, passive torque at DF ROM, and shear elastic modulus.

	HS condition		LL condition	
	PRE	POST	PRE	POST
DF ROM (°)	26.0 ± 9.7	32.1 ± 11.2^*^^,^^#^	24.5 ± 8.1	28.2 ± 8.5^*^
Change (%)	25.7 ± 19.9^†^		16.0 ± 11.8	
Passive torque at DF ROM (Nm)	29.7 ± 13.2	35.6 ± 15.8^*^	29.3 ± 11.2	31.4 ± 12.5^*^
Change (%)	22.9 ± 40.5		6.9 ± 10.8	
Shear elastic modulus of MG (kPa)	21.8 ± 15.7	13.8 ± 8.6^*^	22.8 ± 12.5	13.9 ± 8.4^*^
Change (%)	−38.8 ± 14.5^†^		−22.2 ± 33.8	

HS, high-intensity and short-duration; LL, low-intensity and long-duration; PRE, before stretching intervention; POST, after stretching intervention; DF ROM, dorsiflexion range of motion; MG, medial gastrocnemius; data presented as mean ± standard deviation.

* *p* < 0.05 significant difference between PRE and POST.

# *p* < 0.05 significant difference between POST at high-intensity and short-duration and low-intensity and long-duration.

† *p* < 0.05 significant difference between conditions.

## Discussion

We investigated the effects of high-intensity, short-duration and low-intensity, long-duration of SS with the same total work on DF ROM and shear elastic modulus of MG. Our results showed that DF ROM was increased significantly for both conditions, and DF ROM after the high-intensity and short-duration condition was significantly greater than that after the low-intensity and long-duration condition. Conversely, passive torque at DF ROM changed significantly after both protocols, with no significant difference between the two protocols. Shear elastic modulus was decreased significantly for both conditions, and shear elastic modulus after the high-intensity and short-duration condition was significantly greater than that after the low-intensity and long-duration condition. To the best of our knowledge, our study is the first to investigate the effects of high-intensity and short-duration and low-intensity and long-duration SS with the same impact of intensity and duration including 120% intensity on shear elastic modulus.

Our results showed that DF ROM significantly increased after both high-intensity and short-duration SS and low-intensity and long-duration protocol. The previous studies showed that ROM significantly increased after 120% intensity or 50% intensity of SS – the intensities chosen for this study – and this result was similar to that of previous research (Kataura et al., 2017; Marchetti et al., 2019). In addition, regarding the duration of SS, a previous study reported that ROM significantly increased after 20 s SS (Sato et al., 2020), which supported our results. Therefore, it was assumed that the two SS protocols in this study were effective for increasing ROM.

Interestingly, our study showed that DF ROM at POST in the high-intensity and short-duration protocol was higher than that in the low-intensity and long-duration protocol. Freitas et al. (2015b) showed that a higher intensity of SS was more effective in increasing ROM, which is consistent with our results. Generally, the mechanism of the increase in ROM is the increase in the capacity to tolerate loading prior to stretch termination (the increase in stretch tolerance) and the changes in viscoelastic properties of the muscle-tendon unit (Behm et al., 2016). The results in the current study showed that passive torque at DF ROM was significantly increased and shear elastic modulus was significantly decreased after SS at both protocols. Additionally, the results showed that changes in DF ROM were significantly correlated with passive torque at DF ROM after both protocols. Kay et al. (2016) have reported that a significant positive correlation is observed between the changes (Δ) in DF ROM and passive torque at DF ROM. Moreover, Freitas et al. (2015b) reported that stretch tolerance was significantly modified after only a high-intensity stretching protocol. Therefore, it is possible that the superior change in DF ROM in high-intensity and short-duration protocol than low-intensity and long-duration protocol could be because SS at higher intensity was more effective on stretch tolerance. In fact, we found that Spearman’s rank-order correlation was higher for the high-intensity and short-duration protocol (*ρ* = 0.68, data was not shown) than for the low-intensity and long-duration protocol (*ρ* = 0.60, data was not shown). Thus, our results suggest that the stretching intensity could be more important for changes in DF ROM than stretching duration with the same load of intensity and duration.

This study showed that shear elastic modulus of MG was significantly decreased after both SS protocols. Nakamura et al. (2014) reported a significant correlation between the change in shear elastic modulus and the change in muscle stiffness. Furthermore, in previous studies, the mechanism of the decrease in muscle stiffness could be resulted in the change in connective tissue (Morse et al., 2008; Nakamura et al., 2011). Therefore, in this study, the change in the shear elastic modulus could be contributed to the change in the connective tissue property. On the other hand, in this study, the relative change in shear elastic modulus of MG in the high-intensity and short-duration was greater than the low-intensity and long-duration. Previous study reported that muscle-tendon stiffness at 120% intensity was smaller than that of at 100% intensity (Takeuchi and Nakamura, 2020). On the other hand, previous study showed that shear elastic modulus of MG was significantly decreased after 80% stretching intensity, with no significant decreases after 40 and 60% stretching intensity (Freitas et al., 2015a). Sato et al. (2020) reported that shear elastic modulus was not significantly decreased after 20 s of SS, whereas Nakamura et al. (2014) reported that shear elastic modulus was significantly decreased after 120 s of SS with 100% stretching intensity. Therefore, it is considered that intensity and duration of SS above a certain level is required to decrease shear elastic modulus. However, the relative change in the high-intensity and short-duration was greater than that of the low-intensity and long-duration. Thus, we suggested that stretching intensity was important to decrease muscle stiffness than stretching duration. Recently, the previous studies indicated that the dorsiflexion ROM could be increased immediately and chronically increased after stretching intervention because of the change in sciatic nerve stiffness not muscle stiffness (Andrade et al., 2018, 2020). However, in this study, the change in sciatic nerve stiffness was not measured. Therefore, a comparison of the effects of high-intensity and short-duration or low-intensity and long-duration SS with the same load on the sciatic nerve stiffness is needed.

In this study, our results showed a significant increase in the DF ROM and a decrease in the shear elastic modulus of MG after both high-intensity and short-duration and low-intensity and long-duration SS. Therefore, in order to increase the ROM or decrease the shear elastic modulus, both stretching protocols might be prescribed. However, because high-intensity and short-duration stretching is more effective in changing the ROM and the shear modulus rather than low-intensity and long-duration stretching, it is effective to prescribe high-intensity and short-duration stretching if high-intensity stretching is possible. On the other hand, because high-intensity stretching is painful (Takeuchi and Nakamura, 2020), if high-intensity stretching is not possible due to pain, low-intensity and long-duration stretching could be prescribed to change the ROM and muscle stiffness.

## Conclusion

This study investigated the effect of high-intensity and short-duration or low-intensity and long-duration SS protocols with the same total work on DF ROM and shear elastic modulus. The results showed that DF ROM increased significantly in both protocols, and the increase was greater in the high-intensity and short-duration protocol than in low-intensity and long-duration. Conversely, passive torque at DF ROM (stretch tolerance) increased significantly after SS in both protocols, with no significant difference between the two protocols. On the other hand, shear elastic modulus was decreased significantly for both conditions, and shear elastic modulus after the high-intensity and short-duration condition was significantly greater than that after the low-intensity and long-duration condition. Our results suggest that a higher intensity of SS has greater effects on DF ROM and muscle stiffness even if stretching duration was shorter.

## Data Availability Statement

The raw data supporting the conclusions of this article will be made available by the authors, without undue reservation.

## Ethics Statement

The studies involving human participants were reviewed and approved by the ethics committee at the Niigata University of Health and Welfare, Niigata, Japan. The patients/participants provided their written informed consent to participate in this study.

## Author Contributions

TF, RK, SS, KYh, KYs, RO, and MN: conceptualization, investigation and methodology. TF and MN: data curation. TF: formal analysis, visualization and writing – original draft. MN: funding acquisition, project administration, supervision, writing – review and editing. All authors have read and agreed to the published version of the manuscript.

### Conflict of Interest

The authors declare that the research was conducted in the absence of any commercial or financial relationships that could be construed as a potential conflict of interest.
